# Left Ventricular Mass Index and Relative Wall Thickness Predict Atrial High‐Rate Episodes in Patients With Pacemaker Implantation

**DOI:** 10.1111/echo.70363

**Published:** 2025-12-10

**Authors:** Qin Zhang, Limin Xiang, Gege Zhao, Enbo Zhan, Jiali Tian

**Affiliations:** ^1^ Department of Cardiology The Fifth Affiliated Hospital of Sun Yat‐Sen University Zhuhai China; ^2^ Department of Ultrasound The Fifth Affiliated Hospital of Sun Yat‐Sen University Zhuhai China; ^3^ Medical Insurance Department The Fifth Affiliated Hospital of Sun Yat‐Sen University Zhuhai China

**Keywords:** atrial high‐rate episodes, cardiac implantable electronic devices, left ventricular mass index, relative wall thickness

## Abstract

**Background:**

Previous studies have shown that atrial high‐rate episodes (AHREs) after cardiac device implantation are associated with the occurrence of stroke. This study aimed to explore the predictive value of echocardiographic indices, namely left ventricular mass index (LVMI) and relative wall thickness (RWT), for AHREs following implantation of a cardiac implantable electronic device (CIED).

**Methods:**

This was a single‐center retrospective study. Patients who underwent initial dual‐chamber permanent pacemaker implantation at the Fifth Affiliated Hospital of Sun Yat‐sen University from January 1, 2020 to March 30, 2025, were included. Routine postoperative pacemaker programming was performed, and the occurrence of AHREs was set as the study endpoint. Based on whether AHREs were recorded in pacemaker programming, patients were divided into the AHREs group and non‐AHREs group. Clinical baseline data, serological indicators, and echocardiographic indices were compared between the two groups. Spearman correlation analysis was used to evaluate the correlations between echocardiographic indices and permanent pacemaker implantation parameters (pacemaker threshold, pacemaker sensing, atrial output voltage). Cox regression analysis was conducted to identify independent predictors of AHREs. Receiver operating characteristic (ROC) curve analysis was performed to assess the predictive value of these indices for AHREs. Additionally, Kaplan–Meier survival analysis was used to compare the cumulative incidence of AHREs among groups with different levels of RWT and LVMI.

**Results:**

A total of 122 patients were included, and 45 patients developed AHREs during the follow‐up period. Early diastolic mitral inflow E‐wave velocity (*r* = 0.297, *p* < 0.001) and right atrial parameters (*r* = 0.280, *p* = 0.002) were positively correlated with pacemaker sensing parameters, with statistically significant yet weak correlations. Univariate Cox regression analysis revealed significant correlations between the occurrence of AHREs and early diastolic tricuspid inflow E‐wave velocity, fractional shortening (FS), RWT, diastolic wall strain (DWS), LVMI, and type of cardiac remodeling (all *p* < 0.05). Multivariate Cox regression analysis demonstrated that lower RWT (*B* = −7.576, *p* = 0.007) and higher LVMI (*B* = 0.013, *p* = 0.003) were associated with an increased risk of AHREs. ROC curve analysis showed that the area under the curve (AUC) of LVMI and RWT for predicting AHREs were 0.722 and 0.716, respectively. Kaplan–Meier survival analysis indicated that patients with LVMI  ≥ 114.930 or RWT < 0.392 had a higher incidence of AHREs within 5 years after dual‐chamber permanent pacemaker implantation.

**Conclusion:**

In our study, echocardiographic indices were correlated with permanent pacemaker implantation parameters. LVMI and RWT are independent predictors of AHREs. Patients with LVMI ≥ 114.930 or RWT < 0.392 after cardiac device implantation have a high incidence of AHREs, which provides a basis for early clinical intervention.

## 1 Introduction

Atrial Fibrillation (AF), one of the most prevalent types of arrhythmia in the adult population, represents a major risk factor by significantly increasing the risk of severe cardio‐cerebrovascular complications such as stroke, heart failure, and myocardial infarction [1]. Notably, approximately 30%–40% of AF patients in clinical practice present with occult or subclinical AF [2]. These patients lack typical clinical symptoms such as palpitations and chest tightness, and their abnormal heart rhythms are often difficult to detect using routine short‐term electrocardiogram (ECG) recordings. This leads to delayed diagnosis or even missed diagnosis, thereby leading to missed opportunities for early intervention.

With the widespread application of cardiac implantable electronic devices (CIEDs) in patients with bradyarrhythmias such as sick sinus syndrome and atrioventricular block, the built‐in atrial electrodes enable long‐term and continuous monitoring of atrial electrical activity. This can effectively identify atrial high‐rate episodes (AHREs) that are hard to detect by traditional examination methods. AHREs refer to periods of rapid atrial electrical activity characterized by an atrial rate exceeding 180 beats per minute lasting for ≥ 5 min [3]. Existing studies have confirmed that the incidence of AHREs can be over 30% within 6 months after implantation in pacemaker recipients with no previous history of AF [4, 5], and AHREs have been recognized as an independent risk factor for stroke [6]. Therefore, establishing an effective risk prediction system for AHREs is crucial for enabling early identification and stratification of at‐risk patients after cardiac device implantation, thereby supporting targeted preventive interventions and mitigating long‐term adverse cardiovascular outcomes.

Current clinical practice lacks specific methods for predicting AHREs. Traditional approaches, which rely on clinical symptoms and short‐term ECG, are limited by the occult and paroxysmal nature of AHREs, making early prediction challenging [7]. As a non‐invasive imaging technique, echocardiography can directly assess cardiac structural remodeling (e.g., chamber enlargement, ventricular wall thickening) and functional abnormalities (e.g., systolic and diastolic dysfunction) through quantitative indices. Previous studies have found that echocardiographic indices such as left ventricular mass index (LVMI) and diastolic wall strain (DWS) are closely associated with the occurrence of clinical overt AF and can serve as potential biomarkers for AF risk assessment [8, 9]. However, relevant studies at home and abroad on the association between key echocardiographic indices (such as LVMI and RWT) and the occurrence of AHREs after cardiac device implantation, as well as the interaction between these indices and pacemaker implantation parameters (e.g., sensing, threshold), remain relatively scarce, and no clear conclusions have been formed. This study aims to explore the predictive value of echocardiographic indices for atrial high‐rate events and provide clinical evidence for the management of patients after pacemaker implantation.

## 2 Methods

### 2.1 Study Population

**FIGURE 1:**
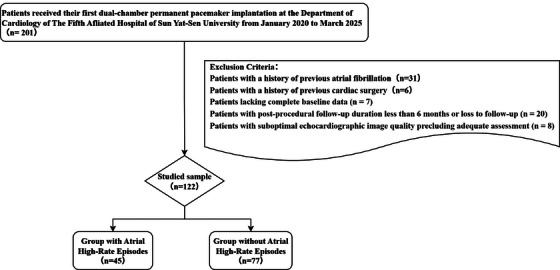
Flowchart of patient selection and inclusion in the study.

This study is a retrospective observational study. This study was approved by the Ethics Committee of the Fifth Affiliated Hospital of Sun Yat‐Sen University (Ethics approval number: 2025 K278‐1), with waiver of written informed consent. The medical records of 122 patients who underwent initial dual‐chamber permanent pacemaker implantation between January 1, 2020, and March 30, 2025, were retrospectively reviewed (Figure 1).

Inclusion Criteria:

1. Diagnosis of sick sinus syndrome or atrioventricular block and implantation of a dual‐chamber permanent pacemaker in accordance with the ACCF/AHA/HRS Guidelines for Device‐Based Therapy of Cardiac Rhythm Abnormalities [10];
2. Aged over 18 years, of any gender, with a complete preoperative electrocardiogram, echocardiography, and laboratory data;
3. The pacemaker was capable of monitoring atrial events, with the atrial electrode positioned in the right atrial appendage;
4. Received regular pacemaker programming for more than 6 months after surgery.

Exclusion Criteria:

1. A history of atrial fibrillation;
2. A history of cardiac electrical cardioversion or catheter ablation;
3. Cardiac function classified as NYHA Class III or higher;
4. Presence of organic heart diseases such as chronic heart failure, myocardial infarction, severe valvular heart disease, and cardiomyopathy;
5. A history of previous cardiac surgery;
6. Hyperthyroidism or hypothyroidism, severe liver and kidney dysfunction, or multiple organ failure;
7. Incomplete data, defined as missing more than 20% of clinical, laboratory, or echocardiographic variables.

### 2.2 Data Collection

Preoperative clinical data were retrospectively retrieved from patient records and included age, sex, height, weight, comorbidities, smoking and alcohol consumption history, clinical symptoms, and serological indicators (glucose, uric acid, creatinine, cholesterol, and brain natriuretic peptide levels). Pacemaker programming parameters were obtained from follow‐up records at 1, 3, 6, and 12 months after implantation and at 6‐month intervals thereafter.

All subjects underwent echocardiography within 1 month before cardiac device implantation, and the examination conformed to the standard views specified in the guidelines of the American Society of Echocardiography (ASE) [11]. All echocardiographic data were independently measured by two echocardiography specialists who were blinded to the clinical data (echocardiography equipment: Philips EPIQ 7C Color Doppler Ultrasound and Ultrasound Probe S5‐1, frequency 1–5 MHz). To assess inter‐observer reliability, the intraclass correlation coefficient (ICC) was calculated for key parameters, showing excellent consistency (ICC > 0.90). In the M‐mode echocardiographic view of the left ventricular long‐axis view, the left ventricular end‐diastolic diameter (LVEDD), left ventricular end‐systolic diameter (LVESD), interventricular septal thickness (IVST), and left ventricular posterior wall thickness (LVPWT) were measured. Functional assessments included left ventricular ejection fraction (EF) measured by Simpson's biplane method, and diastolic function was evaluated using early (E) and late (A) diastolic mitral inflow velocities, E/A ratio, and deceleration time. Left ventricular hypertrophy (LVH) was defined using sex‐specific LVMI thresholds: mild LVH: male LVMI >115 to 130 g/m^2^, female  > 95 to 110 g/m^2^; moderate LVH: male LVMI  > 130 to 150 g/m^2^, female  > 110 to 130 g/m^2^; severe LVH: male LVMI > 150 g/m^2^, female  > 130 g/m^2^, based on guidelines [12]. Cardiac remodeling types were classified as follows: concentric hypertrophy (male LVMI  > 115 g/m^2^, female > 95 g/m^2^, and RWT  > 0.42); eccentric hypertrophy (male LVMI  > 115 g/m^2^, female  > 95 g/m^2^, and RWT  ≤ 0.42); concentric remodeling (male LVMI  ≤ 115 g/m^2^, female  ≤ 95 g/m^2^, and RWT  > 0.42); normal geometry (male LVMI  ≤ 115 g/m^2^, female  ≤ 95 g/m^2^, and RWT  ≤ 0.42) [12]. RWT, DWS, LVMI [11], and myocardial mechano‐energetic efficiency (MEE) [13] were calculated. Heart valve regurgitation (mitral, tricuspid, aortic) was graded as mild, moderate, or severe based on standard criteria: for mitral regurgitation, mild was defined as regurgitant jet area  < 4 cm^2^ or  < 20% of left atrial area, moderate as 20%–40%, and severe as  > 40%; for tricuspid regurgitation, mild was defined as regurgitant jet area  < 5 cm^2^, moderate as 5–10 cm^2^, and severe as  > 10 cm^2^; for aortic regurgitation, mild was defined as jet width  < 25% of left ventricular outflow tract, moderate as 25%–65%, and severe as  > 65% [12].

Cardiac device implantation was performed in accordance with guideline standards. All operators had more than 5 years of experience in pacemaker implantation. Device settings and the positions of the right atrial (RA)/right ventricular (RV) leads were determined by the operators themselves. The CIEDs were manufactured by Biotronik (CIED: Edora 8 DR‐T or Enitra 8 DR‐T; RA lead: Solia S 53; RV lead: Solia S 60) or Medtronic (CIED: A3DR01 or X3D01; RA lead: 5076‐52; RV lead: 5076‐58 or 3830‐69) for the patients. After pacemaker implantation, all patients received regular follow‐up and programming in our hospital, and the device recorded arrhythmic events monitored after implantation. Data on AHREs, including the presence, burden, and duration of episodes that met the predefined criteria (atrial rate > 180 beats per minute with a duration of ≥ 5 min), were retrieved via pacemaker interrogation protocols. Using device‐recorded data and evaluation against the predefined AHRE criteria, patients were stratified into two groups: the non‐AHREs group and the AHREs group.

### 2.3 Statistical Analysis

Data were statistically analyzed using SPSS 26.0 and plotted using GraphPad Prism 8.0. The normality of distribution was assessed using the Kolmogorov‐Smirnov test. Normally distributed variables are presented as mean ± standard deviation and compared using independent samples *t*‐tests, whereas non‐normally distributed variables are expressed as medians (interquartile range) and compared using the Mann–Whitney *U* test. Categorical data are expressed as counts (%), and comparisons between groups were performed using the Chi‐square test. Spearman's correlation analysis was used to evaluate the correlations between echocardiographic indices (including LVMI, RWT, DWS, and early diastolic mitral inflow E‐wave velocity) and pacemaker implantation parameters (pacemaker threshold, pacemaker sensing, atrial output voltage). Cox proportional hazards models were employed to identify factors influencing atrial events. Receiver operating characteristic (ROC) curves assessed the predictive value of various indicators for atrial events. The area under the curve (AUC) value was interpreted using established thresholds: 0.5–0.6, fail; 0.6–0.7, poor; 0.7–0.8, fair; 0.8–0.9, good; and > 0.9, excellent [14]. Kaplan–Meier survival analysis with log‐rank tests compared cumulative atrial event rates across groups. A *p* value  < 0.05 was considered statistically significant. For the interpretation of Pearson correlation coefficients (*r*), the following thresholds were used in this study, in line with Cohen's guidelines [15]: small correlation (*r* = 0.1–0.29), moderate correlation (*r* = 0.3–0.49), and large correlation (*r* ≥ 0.5). Missing values below 20% were handled using multiple imputation by chained equations (MICE) for completely random missing data. This method iteratively established multiple linear regression models to predict missing values and perform imputation.

## 3 Results

All 122 patients (77 males; 45 females) met the inclusion and exclusion criteria. All patients were followed for at least 6 months. During follow‐up, AHRE was detected in 45/122 patients (36.89%). Patients were divided into two groups: the non‐AHREs group (*n* = 77) and the AHREs group (*n* = 45).

### 3.1 Comparison of Clinical Data Between Groups With and Without AHREs

Clinical characteristics of the two groups are summarized in Table 1. There were no statistically significant differences between the two groups in baseline clinical characteristics, medical history, or serological tests (*p* > 0.05).

**TABLE 1:** Comparison of clinical characteristics between groups.

Indicator	non‐AHREs (*n* = 77)	AHREs (*n* = 45)	*^t/Z/χ2^*	*p*
Age (years)	68.19 ± 13.51	69.18 ± 13.79	0.385	0.701
Gender			0.297	0.586
Male	50 (64.9)	27 (60.0)		
Female	27 (35.1)	18 (40.0)		
BSA	1.69 ± 0.16	1.66 ± 0.17	0.882	0.379
BMI	23.58 ± 3.37	23.25 ± 3.46	0.510	0.611
Systolic blood pressure (mmHg)	138.06 ± 22.72	144.73 ± 25.34	1.499	0.137
Diastolic blood pressure (mmHg)	72.74 ± 14.15	73.31 ± 13.51	0.219	0.827
Heart rate (beats/min)	57.81 ± 20	58.56 ± 19.19	0.203	0.840
Smoking history	23 (29.9)	13 (28.9)	0.013	0.909
History of alcohol consumption	15 (19.5)	8 (17.8)	0.054	0.817
Medical history				
Hypertension	41 (53.2)	23 (51.1)	0.052	0.820
Diabetes	15 (19.5)	6 (13.3)	0.753	0.385
Coronary heart disease	17 (22.1)	12 (26.7)	0.330	0.566
Glucose (mmol/L)	5.42 (4.88, 6.13)	5.53 (4.90, 6.41)	−0.852	0.394
Uric acid (µmol/L)	364.10 (298.45, 445.80)	368.00 (308.00, 439.35)	−0.066	0.947
Creatinine (µmol/L)	81.00 (69.30, 93.55)	76.80 (67.70, 94.25)	−0.464	0.642
Total cholesterol (mmol/L)	4.37 ± 1.07	4.15 ± 0.79	1.299	0.197
BNP (pg/mL)	243.94 (118.56, 930.92)	351.09 (146.00, 1791.02)	−1.390	0.164

Abbreviations: BSA, Body Surface Area; BMI, Body Mass Index; BNP, B‐type Natriuretic Peptide.

### 3.2 Comparison of Echocardiographic Parameters Between the Two Groups

Comparison of echocardiographic parameters between the two patient groups is shown in Table 2.

**TABLE 2:** Comparison of echocardiographic parameters between the two groups.

Parameter	non‐AHREs (*n* = 77)	AHREs (*n* = 45)	*^t/Z/^χ^2^*	*p*
Left atrium	37.04 ± 5.86	37.16 ± 5.44	0.109	0.914
EF	68.00 (63.50, 73.00)	70.00 (66.00, 75.00)	−1.634	0.102
Right atrium	36.00 (33.50, 38.00)	35.00 (32.00, 38.00)	−0.834	0.404
Right ventricle	35.00(33.00,37.00)	34.00 (32.00, 36.50)	−0.805	0.421
Interventricular septum thickness	10.00 (9.00, 11.00)	10.00 (9.00, 11.00)	−0.126	0.900
Left ventricular posterior wall thickness (end‐systolic)	14.2 ± 2.73	15.19 ± 2.72	1.921	0.057
Aortic diameter (aortic sinus)	33.00 (30.00, 34.00)	33.00 (30.00, 35.00)	−0.387	0.699
Ascending aorta	32.57 ± 4.08	32.87 ± 4.05	0.387	0.700
Pulmonary artery diameter	22.00 (20.00, 24.00)	22.00 (21.00, 23.50)	−0.345	0.730
Early diastolic mitral valve E‐wave peak velocity	0.78 (0.62, 1.02)	0.81 (0.62, 1.04)	−0.186	0.853
Late diastolic mitral valve orifice peak velocity A	0.87 ± 0.28	0.85 ± 0.28	0.518	0.605
Early diastolic tricuspid inflow E‐wave velocity	**0.54 ± 0.14**	**0.63 ± 0.23**	**2.191**	**0.032**
Late diastolic peak A‐wave velocity at the tricuspid valve	0.44 (0.37, 0.49)	0.42 (0.36, 0.52)	−0.066	0.947
Aortic valve flow velocity during systole	1.35 (1.2, 1.61)	1.31 (1.16, 1.49)	−0.971	0.331
Systolic pulmonary valve flow velocity	**1.02 (0.88, 1.13)**	**0.89 (0.84, 1.03)**	**−2.190**	**0.029**
FS	**40.00 (36.00, 44.00)**	**37.00 (33.00,42.00)**	**−2.158**	**0.031**
RWT	**0.40 (0.36, 0.43)**	**0.36 (0.31,0.39)**	**−3.985**	**0.000**
DWS	**0.37 ± 0.11**	**0.32 ± 0.10**	**2.499**	**0.014**
LVMI	**96.21 ± 23.37**	**127.81 ± 47.1**	**4.209**	**< 0.001**
Left ventricular hypertrophy			6.408	0.093
None	54 (70.1)	24 (53.3)		
Mild	10 (13.0)	6 (13.3)		
Moderate	3 (3.9)	7 (15.6)		
Severe	10 (13.0)	8 (17.8)		
Cardiac remodeling type			10.817	**0.013**
Normal	42 (54.5)	18 (40.0)		
Eccentric hypertrophy	2 (2.6)	9 (20.0)		
Concentric hypertrophy	22(28.6)	12 (26.7)		
Concentric remodeling	11 (14.3)	6 (13.3)		
Myocardial mechanical energy efficiency	1.45 ± 0.58	1.44 ± 0.72	0.030	0.976
Calcification of heart valves	39 (50.6)	18 (40.0)	1.294	0.255
Heart valve regurgitation grade			0.389	0.533
Mild	65 (84.4)	36 (80.0)		
Moderate‐severe	12 (15.6)	9 (20.0)		
Mitral regurgitation			0.009	0.923
None‐minimal	68 (88.3)	40 (88.9)		
Moderate or higher	9 (11.7)	5 (11.1)		
Tricuspid regurgitation			0.071	0.789
None‐minimal	68 (88.3)	39 (86.7)		
Moderate or higher	9 (11.7)	6 (13.3)		
Aortic regurgitation			0.000	1.000
None‐minimal	76 (98.7)	44 (97.8)		
Moderate or higher	1 (1.3)	1 (2.2)		

Abbreviations: AHREs, atrial high‐rate episodes;EF, Ejection Fraction; FS, fractional shortening; RWT, relative wall thickness; DWS, diastolic wall strain; LVMI, left ventricular mass index.

The number of patients with concentric hypertrophy, early diastolic tricuspid inflow E‐wave velocity, and LVMI was significantly higher in the AHREs group than in the non‐AHREs group (*p* < 0.05). Systolic pulmonary valve flow velocity, FS, RWT, and DWS were significantly lower in the AHREs group than in the non‐AHREs group (*p* < 0.05).

### 3.3 Cox Regression Analysis of Factors Influencing Atrial Events

Univariate Cox regression analysis of risk factors for the occurrence of AHREs among all subjects is shown in Table 3. Among 122 subjects, 45 patients experienced AHREs, with a median survival time of 50.00 months (95% Confidence Interval [95% CI]: 29.12–70.88). The state variable was defined as the occurrence of AHREs (yes = 1, no = 0), and follow‐up time served as the time variable. Results demonstrated that early diastolic tricuspid inflow E‐wave velocity, FS, RWT, DWS, LVMI, and cardiac remodeling type were significantly associated with AHREs occurrence (all *p* < 0.05).

**TABLE 3:** Univariate cox regression analysis of factors influencing AHREs.

Indicator	*B*	*SE*	Wald*χ^2^*	*p*	HR (95% CI)
Demographic characteristics					
Age	0.009	0.012	0.569	0.451	1.009 (0.986–1.033)
Gender	0.283	0.311	0.832	0.362	1.327 (0.722–2.44)
BSA	−1.474	0.916	2.589	0.108	0.229 (0.038–1.379)
BMI	−0.043	0.045	0.911	0.340	0.958 (0.876–1.047)
Systolic blood pressure	0.007	0.006	1.605	0.205	1.007 (0.996–1.019)
Diastolic blood pressure	0.002	0.011	0.023	0.879	1.002 (0.980–1.023)
Heart rate	0.002	0.007	0.068	0.794	1.002 (0.987–1.017)
Lifestyle factors					
Smoking history	−0.100	0.331	0.092	0.762	0.905 (0.473–1.730)
Drinking history	−0.234	0.396	0.348	0.555	0.792 (0.364–1.720)
Clinical characteristics					
Hypertension	−0.042	0.298	0.020	0.889	0.959 (0.534–1.722)
Diabetes	−0.206	0.441	0.218	0.640	0.814 (0.343–1.932)
Coronary heart disease	0.160	0.339	0.222	0.638	1.173 (0.603–2.281)
Laboratory parameters					
Glucose	−0.034	0.084	0.163	0.686	0.967 (0.821–1.139)
Uric acid	−0.001	0.001	0.350	0.554	0.999 (0.997–1.002)
Creatinine	−0.004	0.003	1.777	0.183	0.996 (0.989–1.002)
Total cholesterol	−0.211	0.159	1.753	0.185	0.810 (0.593–1.106)
BNP	0.001	0.001	0.253	0.615	1.000 (0.999–1.001)
Echocardiographic measurements					
Left atrium	0.003	0.026	0.011	0.916	1.003 (0.954–1.054)
EF	0.030	0.019	2.596	0.107	1.031 (0.993–1.069)
Right atrium	−0.028	0.033	0.710	0.399	0.972 (0.911–1.038)
Right ventricle	−0.019	0.036	0.272	0.602	0.982 (0.915–1.053)
Interventricular septal thickness	0.096	0.052	3.431	0.064	1.101 (0.994–1.218)
Left ventricular posterior wall thickness (end‐systolic)	0.056	0.051	1.196	0.274	1.057 (0.957–1.168)
Aortic diameter (aortic sinus)	0.049	0.041	1.392	0.238	1.050 (0.968–1.139)
Ascending aorta	0.020	0.036	0.312	0.577	1.020 (0.951–1.095)
Pulmonary artery diameter	−0.003	0.048	0.004	0.952	0.997 (0.907–1.096)
Early diastolic mitral valve E‐wave peak velocity	−0.437	0.535	0.668	0.414	0.646 (0.226–1.843)
Late diastolic mitral valve A‐wave peak velocity	−0.137	0.518	0.070	0.792	0.872 (0.316–2.407)
**Early diastolic tricuspid inflow E‐wave velocity**	**1.723**	**0.666**	**6.689**	**0.010**	**5.599 (1.518**–**20.652)**
Peak velocity of A wave at late diastolic tricuspid valve opening	−0.357	1.064	0.112	0.737	0.700 (0.087–5.635)
Systolic aortic valve orifice flow velocity	−0.113	0.482	0.055	0.815	0.893 (0.347–2.296)
Systolic pulmonary valve flow velocity	−1.095	0.770	2.021	0.155	0.335 (0.074–1.514)
FS	−**0.041**	**0.018**	**5.403**	**0.020**	**0.960 (0.927**–**0.994)**
RWT	−**10.167**	**2.639**	**14.839**	**0.000**	**0.000 (0.000**–**0.007)**
DWS	−**2.845**	**1.326**	**4.603**	**0.032**	**0.058 (0.004**–**0.782)**
LVMI	**0.015**	**0.003**	**25.254**	**0.000**	**1.016 (1.009**–**1.022)**
Left ventricular hypertrophy			3.870	0.276	
None	—	—	—	—	*ref*
Mild	0.438	0.458	0.914	0.339	1.549 (0.632–3.801)
Moderate	0.796	0.434	3.366	0.067	2.217 (0.947–5.190)
Severe	0.370	0.414	0.796	0.372	1.447 (0.642–3.260)
Cardiac remodeling type			9.654	0.022	
Normal	—	—	—	—	*ref*
Concentric hypertrophy	**1.257**	**0.413**	**9.250**	**0.002**	**3.515 (1.564**–**7.903)**
Eccentric hypertrophy	0.213	0.377	0.320	0.572	1.237 (0.591–2.589)
Concentric remodeling	0.229	0.481	0.227	0.634	1.257 (0.49–3.228)
Myocardial mechanical energy efficiency	−0.039	0.243	0.026	0.871	0.961 (0.597–1.547)
Valvular calcification	−0.291	0.308	0.895	0.344	0.748 (0.409–1.366)
Degree of valve regurgitation					
Mild	—	—	—	—	*ref*
Moderate to severe	0.319	0.378	0.713	0.398	1.376 (0.656–2.884)
Mitral regurgitation					
Mild	—	—	—	—	*ref*
Moderate to severe	0.105	0.479	0.048	0.827	1.111 (0.434–2.842)
Tricuspid regurgitation					
Mild	—	—	—	—	*ref*
Moderate to severe	0.318	0.445	0.510	0.475	1.374 (0.575–3.285)
Aortic regurgitation					
Mild	—	—	—	—	*ref*
Moderate to severe	0.569	1.016	0.314	0.575	1.767 (0.241–12.952)

Abbreviations: AHREs, atrial high‐rate episodes;BSA, Body Surface Area; BMI, Body Mass Index;BNP, B‐type Natriuretic Peptide;EF, Ejection Fraction;FS, fractional shortening; RWT, relative wall thickness; DWS, diastolic wall strain; LVMI, left ventricular mass index.

Multivariate Cox regression analysis of risk factors for AHREs occurrence among all subjects is shown in Table 4. Using occurrence of AHREs as the state variable (yes = 1, no = 0), follow‐up time as the time variable, and early diastolic tricuspid inflow E‐wave velocity, FS, RWT, DWS, LVMI, and cardiac remodeling type as independent variables, a Cox proportional hazards model was constructed by entering all variables simultaneously. The multivariate Cox regression model showed that LVMI was a risk factor for AHREs (Hazard Ratio [HR] = 1.013, 95%CI = 1.005–1.022, *p* = 0.003). In contrast, RWT was a protective factor for AHREs (HR = 0.801, 95% CI = 0.682–0.942, *p* = 0.007)

**TABLE 4:** Multivariate cox regression analysis of factors influencing AHREs.

Indicator	*B*	*SE*	Wald*χ^2^*	*p*	HR (95% CI)
Early diastolic tricuspid inflow *E*‐wave velocity	1.102	0.822	1.799	0.180	3.01 (0.602–15.058)
FS	−0.032	0.019	2.885	0.089	0.968 (0.933–1.005)
RWT	−7.576	2.815	7.242	**0.007**	0.801 (0.682–0.942)
DWS	−2.507	1.471	2.904	0.088	0.082 (0.005–1.457)
LVMI	0.013	0.004	9.016	**0.003**	1.013 (1.005–1.022)
Cardiac myocardial remodeling type			1.411	0.703	
Normal	—	—	—	—	*ref*
Concentric hypertrophy	0.370	0.491	0.567	0.451	1.447 (0.553–3.786)
Eccentric hypertrophy	−0.131	0.420	0.097	0.756	0.878 (0.385–2.000)
Concentric remodeling	0.326	0.498	0.430	0.512	1.386 (0.522–3.678)

Abbreviations: AHREs, atrial high‐rate episodes;FS, fractional shortening; RWT, relative wall thickness; DWS, diastolic wall strain; LVMI, left ventricular mass index.

### 3.4 ROC Curve Analysis of RWT and LVMI for Predicting AHREs

**FIGURE 2:**
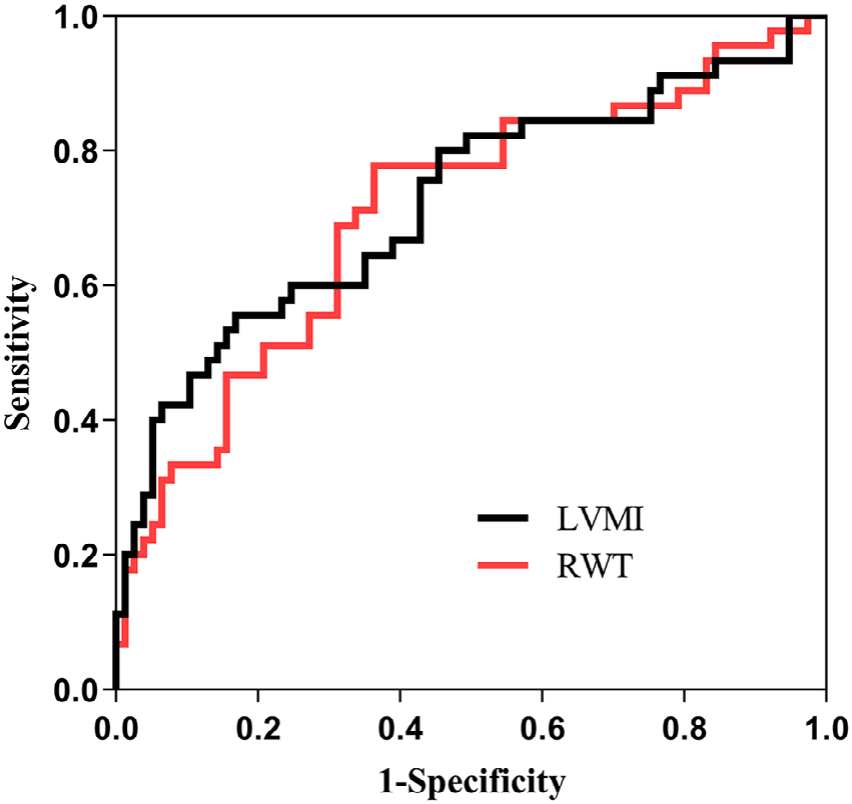
ROC curve analysis for RWT and LVMI in predicting AHREs. AHREs, atrial high‐rate episodes; LVMI, left ventricular mass index; ROC, receiver‐operating characteristic; RWT, relative wall thickness.

Results of ROC analysis of RWT and LVMI in predicting AHREs are shown in Table 5, with the corresponding ROC curves presented in Figure 2. The AUC values for LVMI and RWT in predicting AHREs were 0.722 and 0.716, respectively, indicating fair predictive ability. A RWT value of 0.392 was identified as the cutoff for predicting AHREs, with a sensitivity of 77.8% and specificity of 63.6%. Similarly, for LVMI, a cutoff of 114.930 was associated with a sensitivity of 55.6% and specificity of 83.1%.

**TABLE 5:** ROC curve analysis of RWT and LVMI for predicting AHREs.

Parameter	LVMI	RWT
AUC	0.722	0.716
95% CI	0.623–0.820	0.620–0.813
*p*	**< **0.001	**< **0.001
Threshold	114.930	0.392
Sensitivity	55.6	77.8
Specificity	83.1	63.6

Abbreviations: ROC, receiver‐operating characteristic; RWT, relative wall thickness; LVMI, left ventricular mass index;AHREs, atrial high‐rate episodes;AUC, area under the curve.

### 3.5 Comparison of Cumulative Atrial Event Incidence Rates across Different RWT and LVMI Groups

**FIGURE 3:**
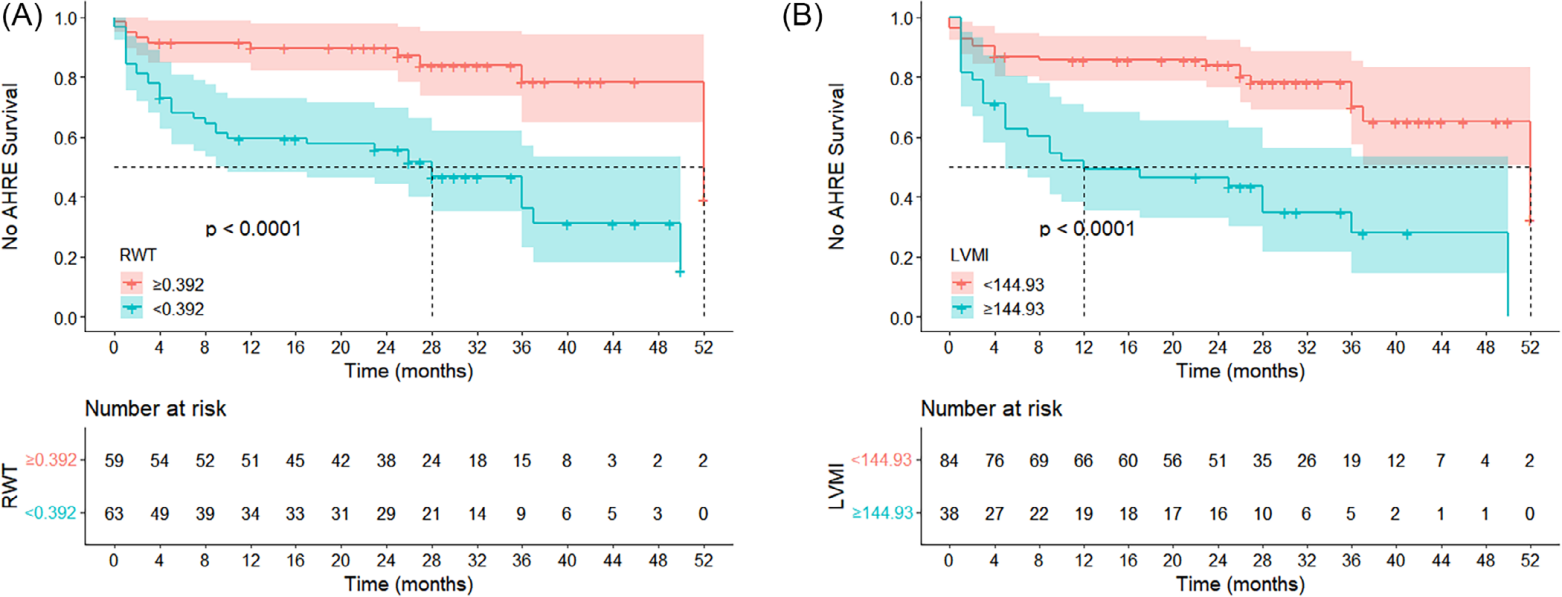
Cumulative incidence of AHREs in the RWT and LVMI groups. (A) cumulative incidence of AHREs in the RWT groups; (B) cumulative incidence of AHREs in the LVMI groups. AHREs, atrial high‐rate episodes; LVMI, left ventricular mass index; RWT, relative wall thickness.

The cumulative incidence rates of AHREs across different groups stratified by RWT and LVMI are shown in Table 6. Meanwhile, the cutoff values, determined via the Youden index from ROC curves, are presented in Figure 3. Log‐rank test comparisons revealed that a significantly higher cumulative incidence of AHREs in the RWT < 0.392 group compared to the ≥ 0.392 group (*p* < 0.05), and similarly, a significantly higher cumulative incidence of AHREs in the LVMI ≥ 114.930 group compared to the < 114.930 group (*p* < 0.05).

**TABLE 6:** Comparison of cumulative AHREs rates across different RWT and LVMI groups.

Indicator	Number of cases	Median survival time (95% CI)	Log‐rank	*p*
LVMI			21.200	**<**0.001
**< **114.930	84	52.00 (30.89, 73.11)		
≥ 114.930	38	12.00 (0.00, 29.47)		
RWT			19.798	**<**0.001
< 0.392	63	28.00 (19.07, 36.93)		
≥ 0.392	59	52.00 (29.63, 74.37)		

Abbreviations: AHREs, atrial high‐rate episodes;RWT, relative wall thickness; LVMI, left ventricular mass index.

### 3.6 Correlation Analysis Between Echocardiographic Parameters and Pacemaker Implantation Parameters

**FIGURE 4:**
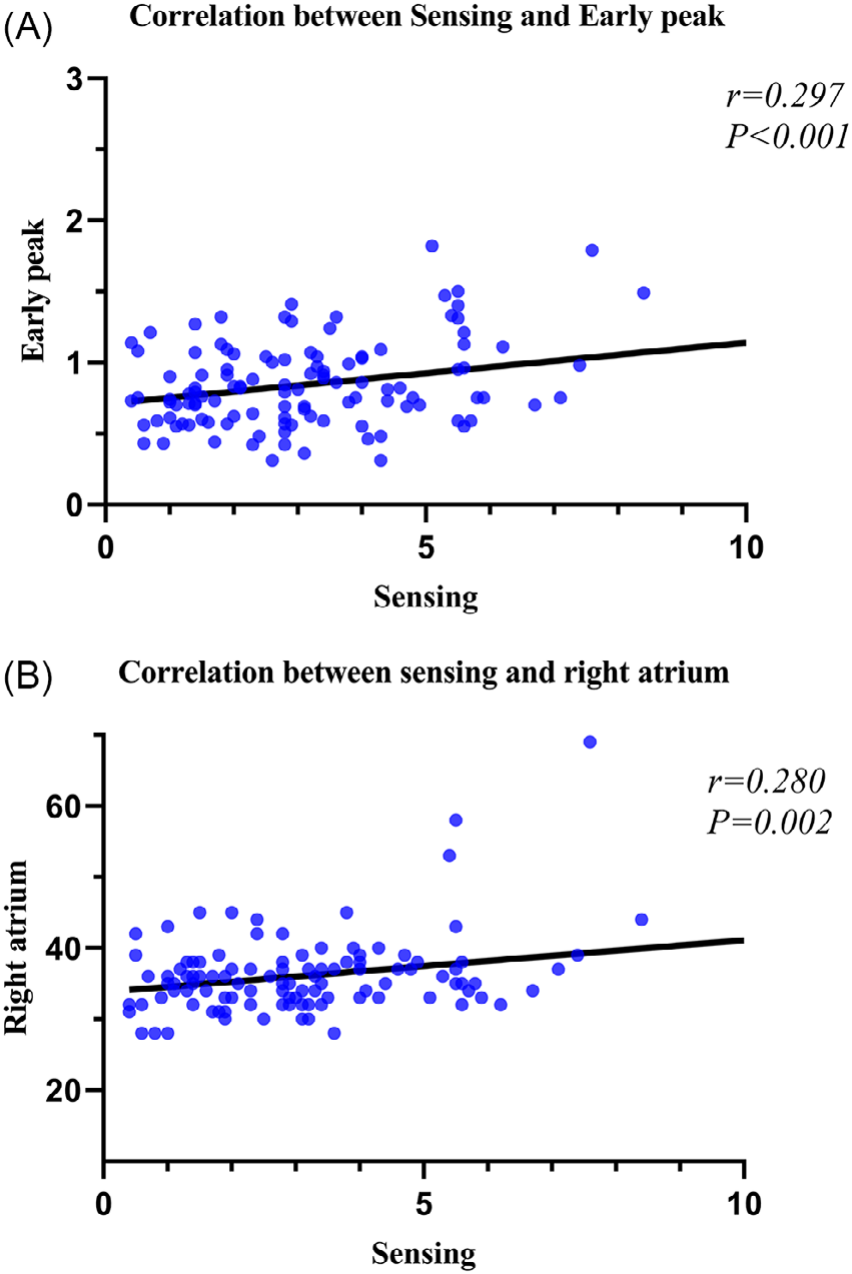
Correlation analysis of echocardiographic parameters with atrial threshold, atrial sensing, and atrial output voltage. (A) correlation between sensing and early peak; (B) correlation between sensing and right atrium.

Results of correlation analysis between echocardiographic parameters and pacemaker implantation parameters are shown in Table 7. Early diastolic mitral valve E‐wave velocity (*r* = 0.297, *p* < 0.001) and right atrial velocity (*r* = 0.280, *p* = 0.002) showed statistically significant correlations with P/R wave, exhibiting small positive correlations (Figure 4).

**TABLE 7:** Correlation analysis of echocardiographic parameters with atrial threshold, atrial sensing, and atrial output voltage.

		RWT	LVMI	Early diastolic mitral valve E peak velocity	Right atrium
Atrial threshold	*r*	0.022	−0.152	−0.015	−0.154
	*P*	0.807	0.095	0.868	0.095
Atrial sensing	*r*	−0.077	0.093	**0.297**	**0.280**
	*p*	0.399	0.309	**< 0.001**	**0.002**
Atrial output voltage	*r*	−0.029	−0.058	0.017	0.055
	*p*	0.755	0.527	0.858	0.557

Abbreviations: RWT, relative wall thickness; LVMI, left ventricular mass index.

## 4 Discussion

In our study, we found that LVMI and RWT are independent predictors of AHREs after dual‐chamber pacemaker implantation. Patients with LVMI ≥ 114.930 or RWT < 0.392 had a significantly increased risk of AHREs within 5 years postoperatively. These findings provide a basis for early clinical intervention using routinely available echocardiographic indices.

Comparison of clinical data between groups with and without AHREs revealed no significant differences in baseline characteristics, medical history, or serological tests (*p *> 0.05). This suggests that traditional risk factors may not adequately predict AHREs, highlighting the need for novel biomarkers such as echocardiographic indices. The absence of differences reinforces that AHREs are often occult and not captured by conventional clinical assessment [7].

In contrast, echocardiographic parameters showed significant differences between groups. The AHREs group exhibited higher early diastolic tricuspid inflow E‐wave velocity, LVMI, and prevalence of concentric hypertrophy, but lower systolic pulmonary valve flow velocity, FS, RWT, and DWS (*p* < 0.05). These findings indicate that structural and functional cardiac abnormalities, particularly left ventricular remodeling and diastolic dysfunction, are associated with AHREs development. Multivariate Cox regression analysis identified RWT < 0.392 (*B* = −7.576, *p* = 0.007) and LVMI ≥ 114.930(*B* = 0.013, *p* = 0.003) as independent predictors of AHREs. ROC analysis confirmed their predictive value, with AUCs of 0.716 and 0.722 for RWT and LVMI, respectively.

Previous studies have shown that among various echocardiographic parameters, RWT is the most effective echocardiographic measurement for assessing the risk of ventricular tachycardia. A decrease in RWT is associated with an increased risk of arrhythmias such as ventricular tachycardia and ventricular fibrillation in heart failure patients [9]. Additionally, RWT is negatively correlated with adverse cardiovascular events after myocardial infarction [16]. This study further expands its application scenario, confirming that RWT < 0.392 can serve as a risk threshold for AHREs after pacemaker implantation.

Regarding LVMI, as a quantitative indicator of left ventricular hypertrophy adjusted for body surface area, it has been proven to be associated with new‐onset AF after aortic valve replacement [8], adverse prognosis after myocardial infarction [17], and mortality risk in heart failure patients [18]. This study, for the first time, links LVMI to AHREs after CIED implantation, revealing that each 1‐unit increase in LVMI was associated with a significant elevation in AHREs risk (*B* = 0.013, *p* = 0.003), and a threshold of 114.930 can effectively identify high‐risk populations. This provides a new perspective for predicting device‐monitored arrhythmias using basic echocardiographic parameters.

The correlation analysis further revealed small but significant associations between echocardiographic parameters (early diastolic mitral E‐wave velocity and right atrial velocity) and pacemaker sensing (*r* = 0.297 and *r* = 0.280, respectively), suggesting a potential interaction between cardiac structure and device electrophysiology.

The mechanisms by which LVMI and RWT predict AHREs can be explained by the “structural remodeling‐electrical remodeling” cascade. Elevated LVMI indicates left ventricular hypertrophy, while abnormal RWT reflects imbalanced ventricular wall thickness‐—both signifying left ventricular remodeling. This pathological change increases electrical conduction heterogeneity, leading to potential breakdown and compensatory atrial electrical remodeling. These findings align with previous studies linking ventricular remodeling to atrial arrhythmogenesis [19, 20, 21].

The clinical implications of our study are significant. LVMI and RWT, obtained through routine echocardiography, offer a non‐invasive and accessible means for risk stratification post‐pacemaker implantation. Patients with high LVMI or low RWT may benefit from enhanced monitoring, early anticoagulation assessment, or interventions targeting ventricular remodeling.

Our study has several limitations. First, it is a single‐center retrospective study, which is subject to inherent biases. Second, previous studies have found an association between DWS and AHREs [22]. In our study, the DWS in the event group was significantly lower than that in the non‐AHREs group in the comparison of general echocardiographic parameters. However, no significant correlation was observed between DWS and AHREs in the multivariate analysis, which may be attributed to the small sample size.

To address the above limitations, future studies may adopt a “multi‐center prospective cohort” design, expand the sample size, and extend the follow‐up period (e.g., beyond 5 years) to focus on exploring the combined predictive value of LVMI, RWT, and DWS. Meanwhile, interventional studies can be conducted to verify whether early interventions (such as drugs to improve ventricular remodeling and intensive anticoagulation) targeting the population with “LVMI ≥ 114.930 or RWT < 0.392” can reduce the risk of AHREs and subsequent stroke, ultimately translating basic research conclusions into clinical practice guidelines.

## 5 Conclusions

LVMI and RWT, which reflect left ventricular structural remodeling, are closely associated with AHREs after dual‐chamber pacemaker implantation and serve as independent predictors of AHREs. Patients with postoperative LVMI ≥ 114.930 or RWT < 0.392 have a significantly increased risk of AHREs within 5 years. Clinically, targeted interventions (such as treatments to improve ventricular remodeling, enhanced arrhythmia monitoring, or individualized anticoagulation assessment) should be initiated as early as possible to reduce the risk of long‐term stroke and adverse cardiovascular events.

## Ethics Statement

This clinical study was conducted by the Declaration of Helsinki and Chinese clinical research norms and regulations. The study was approved by the Ethics Committee of the Fifth Affiliated Hospital of Sun Yat‐Sen University (Ethics approval number: 2025 K278‐1), with a waiver of written informed consent due to retrospective noninterventional design. All patient data were maintained with confidentiality. This research followed the observational cohort guideline.

## Conflicts of Interest

The authors declare no conflicts of interest.

## Data Availability

The datasets generated or analyzed during the study are available from the corresponding author on reasonable request.
